# Better Together: 14-Month-Old Infants Expect Agents to Cooperate

**DOI:** 10.1162/opmi_a_00115

**Published:** 2024-02-01

**Authors:** Liza Vizmathy, Katarina Begus, Gunther Knoblich, György Gergely, Arianna Curioni

**Affiliations:** Department of Cognitive Science, Central European University, Vienna, Austria; Department of Psychology, University of Copenhagen, Copenhagen, Denmark; Institute of Computer Technology, Technische Universität Wien, Vienna, Austria

**Keywords:** cooperation, infant cognition, naive utility, joint action

## Abstract

Humans engage in cooperative activities from early on and the breadth of human cooperation is unparalleled. Human preference for cooperation might reflect cognitive and motivational mechanisms that drive engagement in cooperative activities. Here we investigate early indices of humans’ cooperative abilities and test whether 14-month-old infants expect agents to prefer cooperative over individual goal achievement. Three groups of infants saw videos of agents facing a choice between two actions that led to identical rewards but differed in the individual costs. Our results show that, in line with prior research, infants expect agents to make instrumentally rational choices and prefer the less costly of two individual action alternatives. In contrast, when one of the action alternatives is cooperative, infants expect agents to choose cooperation over individual action, even though the cooperative action demands more effort from each agent to achieve the same outcome. Finally, we do not find evidence that infants expect agents to choose the less costly alternative when both options entail cooperative action. Combined, these results indicate an ontogenetically early expectation of cooperation, and raise interesting implications and questions regarding the nature of infants’ representations of cooperative actions and their utility.

## INTRODUCTION

The scope and breadth of cooperative activities that humans engage in is unparalleled in any other species. Although various non-human animal species also engage in some cooperative and prosocial activities, the variety and breadth of human social cooperation seem to differ from these in qualitatively significant ways. Not only do humans start to spontaneously engage in cooperative activities early in development (Warneken et al., 2012; Warneken & Tomasello, 2007), they also prefer to achieve their goals by acting together with a partner rather than alone (Rekers et al., 2011). Importantly, children show a preference to engage with collaborative partners to pursue a goal together even in situations where cooperation is neither necessary nor the most efficient action alternative available to achieve their goal (Rekers et al., 2011).

In contrast, while chimpanzees, our closest evolutionary relatives, are able to perform cooperative activities, they only seem to do so when it is instrumental for achieving their goal, that is, when the desired outcome could not be achieved by a single individual alone, or when cooperation offers greater rewards (Bullinger et al., 2011; Rekers et al., 2011). Cooperation and prosocial acts are uncommon in most great apes: food transfer is rare even among kin; teaching and allo-maternal care are virtually absent (Hrdy, 2009; Jaeggi et al., 2010). These kinds of cooperative social behaviours are more commonly observed in cooperative breeders like new world monkeys (Brotherton et al., 2001; Burkart & van Schaik, 2010). However, prosocial acts in these species are typically carried out by adult individuals only.

Unlike other great apes, and similarly to new world monkeys, humans evolved as cooperative foragers and breeders for whom acting together with conspecifics provides an essential survival benefit. By cooperating in a variety of domains, humans can achieve highly valuable goals that would be otherwise unavailable. The survival advantage that resulted from the social interdependence of humans in foraging and the necessity to engage in cooperative activities flexibly and quickly may have constituted a crucial pressure for the evolution and selection of cognitive and motivational mechanisms reinforcing a preference for cooperation (Tomasello et al., 2012). It has also been proposed that the increasing complexity and scale of human cooperation necessitated the cooperative abilities to emerge earlier in the development, allowing more time for the development of skills necessary for individuals to become proficient cooperators by adulthood, and thereby increasing their fitness (Tomasello & Gonzalez-Cabrera, 2017). It has been also proposed that human infants’ cooperative and communicative abilities have evolved in the context of cooperative breeding, prevalent in human groups (Hawkes, 2014; Hrdy, 2009, 2016). As cooperative child-rearing allowed women to have more closely spaced offspring and to distribute and share caregiving tasks with others, infants faced the new challenge of competing with siblings and other children for the attention of multiple caregivers. Infants who could better understand and predict the behaviour of the adults could also solicit their help and attention more successfully. This may have constituted a significant evolutionary pressure for the early onset of social cognition, communication, and various cooperative skills in humans (Herrmann et al., 2007).

Experimental evidence from developmental psychology shows that human infants expect agents to cooperate selectively. At the age of 17 months, they expect members of the same group to provide help to each other, but not to the members of other groups (Jin & Baillargeon, 2017). At 6 months of age infants do not seem to expect agents to help others when the group belonging is not specified (Hamlin et al., 2007; see Schlingloff et al., 2020 for similar results with 15 months old). Additionally, from the age of 14th months start to engage in a variety of cooperative behaviors themselves, such as helping an adult by handing them the object the adult is unsuccessfully reaching for (Warneken & Tomasello, 2007). Already by the age of 18 months, infants perform coordinated cooperative activities requiring infants to complement the actions of a partner to achieve a common goal (Warneken et al., 2006). By 21 months, toddlers encourage their cooperative partner to re-engage the joint activity if the partner stops interacting (Warneken et al., 2012). Not only are young children *able* to cooperate with others, but they seem to be highly motivated to do so. Rekers et al. (2011) compared the behaviour of 3-year-old children and chimpanzees when facing a task in which they could obtain food by either acting together with a conspecific, or by acting alone. Results showed that children, but not chimpanzees, prefer to coordinate with a partner as opposed to obtaining food on their own. Importantly, 3-year-olds preferred to collaborate even when acting together with a partner did not represent a more efficient means to achieve their goal than acting alone.

We hypothesise that if humans have dedicated cognitive and motivational mechanisms supporting the propensity to cooperate (Herrmann et al., 2007), young infants, even when their cooperative engagement and abilities are still limited, may hold expectations about other agents’ preferences for cooperative activities as a part of their developing cooperative abilities. The aim of the current studies was to investigate whether infants indeed hold expectations about preference for cooperation in others, and whether the utility calculus framework can explain such expectations. Here and thereafter, we use the term ‘expect’ when referring to infant participants, their behavior, and looking time measures. We reserve the term ‘prefer’ for the observed agents and their hypothetical mental state, presumed to be assigned by observers, including infants, as a motivational explanation for the actions of others. We tested whether 14-month-old infants expect other agents to choose non-mutualistic cooperation over individual goal achievement, that is, to choose cooperation even when it is not instrumentally necessary to achieve the goal. Furthermore, we investigated whether infants apply utility calculations to assess instrumental costs in individual and joint actions.

To address this question, we built on the body of previous results which has demonstrated infants’ early sensitivity to the rationality and utility of goal-directed actions (Gergely & Csibra, 2003; Jara-Ettinger et al., 2015; Liu et al., 2017). Even before their first birthday, human infants have been shown capable of understanding goals and preferences of other agents by applying and combining a wide range of social information available to them, such as intentions (Woodward, 1998) and mental states (Gergely et al., 1995; Luo & Baillargeon, 2007; Luo & Johnson, 2009; Onishi & Baillargeon, 2005), together with physical properties of agents and the environment (Saxe et al., 2006). Most importantly, this converging body of evidence supports the theory that infants apply a ‘teleological stance’ to observed actions (Gergely & Csibra, 2003), that is an action interpretation system implementing the basic assumption that agents will choose the most efficient means available to achieve their goals. As early as 3 months of age, infants are able to predict an agent’s goal by assuming she would achieve it by incurring minimal costs (Liu et al., 2019; Skerry et al., 2013), and 9-month-old infants do not attribute goals to agents whose actions unfold inefficiently (Begus et al., 2020; Hernik & Southgate, 2012; Southgate & Csibra, 2009).

The Naive Utility Calculus model (Jara-Ettinger et al., 2015) provides a recent computational framework which further expands the teleological stance. According to this model, one can not only predict an agent’s choice based on the relative costs of the action alternatives available to the agent, but also infer the agent’s preference for a given goal, based on how costly is the action performed to achieve it. Experimental work has provided evidence that infants indeed seem to apply these principles when interpreting others’ actions. In action observation paradigms, 6-month-old infants expected agents to minimize the costs of their actions, and 10-month-old infants inferred an agent’s preference between two goals, based on the costs they incurred to achieve each of them (Liu et al., 2017; Liu & Spelke, 2017). Capitalising on infants’ precocious abilities to infer agents’ individual goals and preferences based on observed actions, we tested infants’ expectations about cooperative joint actions, that is, actions in which two agents coordinate in space and time to jointly bring about a change in the environment (Sebanz et al., 2006).

Cooperative joint actions integrate the principles of goal directed instrumental actions with cooperation and action coordination between two or more agents. Although little is known about whether and how adults represent the utility of cooperative actions, recent work demonstrated that when they are involved in coordinated joint actions with a partner, adults consistently prioritize joint efficiency over individual efficiency (Török et al., 2019, 2021). This suggests that in the context of a joint action where individuals share a goal, adults are capable and willing to minimize joint costs. Building on research demonstrating that infants expect agents to maximise their individual utility (rewards they obtain minus the costs they incur to achieve a certain goal), here we investigate whether they would hold such expectations even when individual actions are part of a joint action.

One way that the naive utility calculus may be applied in a joint action scenario, is by calculating the separate utilities of the agents’ actions independently, whereby infants’ cost-reward analysis of cooperative activities would equate to the sum of the instrumental utilities of the separate agents. Such a mechanism should result in infants’ expecting cooperative actions only when cooperation is mutually beneficial for the agents involved. Alternatively, infants may expect agents’ behavior to be guided by a preference for cooperation, even when cooperation brings no additional benefits, as may be predicted based on the evolutionary accounts of human propensity to cooperate, reviewed above. If this is the case, a preference for cooperation may be integrated in infants’ utility calculus, by assigning an additional reward (or a reduced cost) to acting cooperatively. Infants’ cost-reward computations in joint action scenarios could therefore not be reduced to the sum of the costs and rewards of the individual agents’ instrumental actions, and additional cooperation-specific variables would have to be inferred. Another possibility is that infants expect agents to prefer cooperation over individual goal achievement but do so without integrating such a preference within instrumental utility computations. Although adults demonstrate the ability to maximize joint utility in cooperative actions, it is possible that this ability develops only later in life, and infants’ expectations are driven by other mechanisms or heuristics. We explore these alternative hypotheses in the studies presented below.

**Figure 1.**
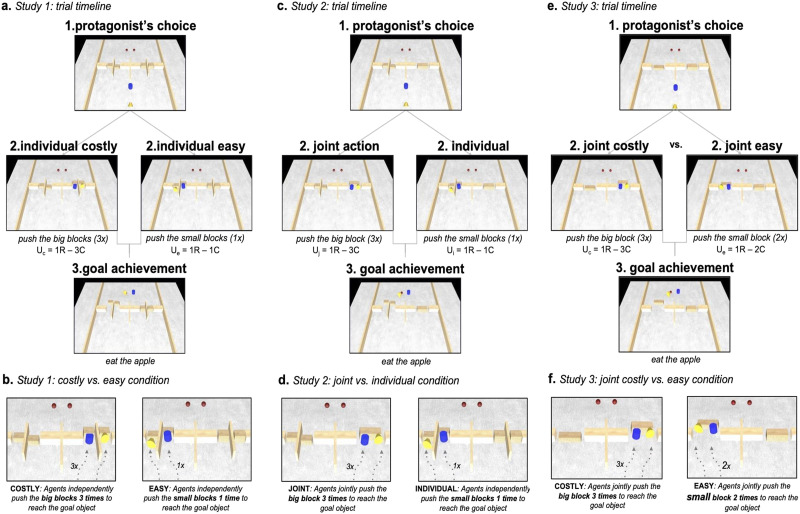
**(A) Trial timeline of costly and easy conditions of Study 1.** At the beginning of each trial, the protagonist agent (yellow) chooses which path to take (costly/easy) to reach the goal object (red apple) (1). The protagonist then moves towards the chosen action alternative, and the second agent follows. Upon reaching the blocks, agents push them away (2), to finally reach the goal objects and eat them (3). Calculations of the observable utility (U), in terms of reward (R) and costs (C) computations of each action, are specified in the figure captions (2). **(B) Close up of costly and easy conditions of Study 1.** From the left, each agent in the costly condition performs 3 pushes to remove the obstacles, 30% bigger in volume compared to the obstacles in the easy condition. Each agent in the easy condition performs 1 push to remove the small obstacles. Onset and offset of two agents’ actions are de-synchronized by 1 second in both conditions. **(C) Trial timeline of joint and individual conditions of Study 2.** At the beginning of each trial, the protagonist agent (yellow) chooses which path to take (joint/individual) to reach the goal object (red apple) (1). The protagonist then moves towards the chosen action alternative, and the second agent follows. After reaching the blocks (or single block in the joint condition), agents push them away (2), to finally reach the goal objects and eat them (3). Calculations of the observable utility (U), in terms of reward (R) and costs (C) computations of each action, are specified in the figure captions (2). **(D) Close up of joint and individual conditions of Study 2.** From the left, each agent in the joint condition performs a short push from the sides, after which both agents perform 2 pushes simultaneously to remove the single obstacle (block), 30% bigger in volume compared to the sum of the two obstacles in the individual condition. Each agent in the individual condition performs 1 push to remove the small obstacle (block). Onset and offset of the two individual agents’ actions were de-synchronized so that actions were not performed simultaneously. **(E) Trial timeline of joint costly and joint easy conditions of Study 3.** At the beginning of each trial, the protagonist agent (yellow) chooses which path to take (joint costly/joint easy) to reach the goal object (red apple) (1). The protagonist then moves towards the chosen action alternative, and the second agent follows. After reaching the block on either side, agents push them away (2), to finally reach the goal objects and eat them (3). Calculations of the observable utility (U), in terms of reward (R) and costs (C) computations of each action, are specified in the figure captions (2). **(F) Close up of joint costly and joint easy conditions of Study 3.** From the left, each agent in the joint costly condition performs a short push from the sides, after which both agents perform 3 pushes simultaneously to remove the single obstacle (block), 30% bigger in volume compared to the obstacle in the easy condition. Both agents in the joint easy condition perform 2 pushes simultaneously to remove the smaller obstacle (block).

We tested 14-month-old infants, who have been shown to understand that actions performed by cooperative partners are complementary and critically interrelated for the achievement of the shared goal (Henderson & Woodward, 2011). In our studies, we presented three groups of infants with animated videos depicting two agents facing two action alternatives, which differed in the costs that the agents would have to incur, but ultimately led to the same rewards (see Figure 1). The different action costs were operationalized by placing different sized obstacles (blocks) that were blocking the agents’ access to their rewards, along the two alternative routes. The obstacles blocking the passage through the two alternative routes differed in volume (and therefore number of actions required to move them out of the way) as well as in whether they could be acted upon jointly (a single larger block) or individually (two smaller separate blocks). In the beginning of each trial the protagonist agent had to decide which path to take towards the goal objects, whereas the second agent always followed the path chosen by the protagonist. The choice performed by the first agent was a crucial part of the experimental manipulation, which allowed us to test infants’ expectations about the agents’ preferences, rather than infants’ own preferences.

Study 1 (*N* = 24) was performed to test whether infants would show sensitivity to instrumental costs based on the volume of the obstacles and the number of actions taken (see Figure 1A). The protagonist agent had to choose between two alternative routes, both requiring each agent to move their own obstacle out of the way. The obstacles were either large blocks that required each agent to perform three actions (Costly condition) or small blocks, which could be moved with a single action (Easy condition, see Figure 1B). According to the naïve utility calculus, infants should expect agents to incur minimal costs in pursuit of a goal, and therefore we predicted that choosing the more costly action alternative in Study 1 would violate infants’ expectations.

Study 2 (*N* = 24) then addressed the critical question. In a nearly identical scenario to Study 1, agents faced two alternative routes, with identical costs and rewards as in Study 1, however with one crucial difference: the costly route of Study 2 involved a single large obstacle and thus allowed the two agents to work cooperatively (Joint condition, see Figure 1C). The large obstacle in Joint condition was identical to the combined volume of the two large obstacles in Costly condition of Study 1 and required 3 actions on behalf of each agent. The alternative route was identical to the Easy condition of Study 1 and thus contained two smaller obstacles, which the agents could each move on their own with a single action (Individual condition, see Figure 1D). Importantly, all the individual movements of agents and position of agents relative to each other were identical in the respective conditions (Costly/Joint; Easy/Individual) in the two studies, both in low level kinematics and in goal structure. Considered from the standpoint of instrumental costs and rewards alone, the conditions of Study 1 and Study 2 are identical and therefore yield the same results: infants should expect agents to take the route requiring fewest actions to reach the rewards. However, if infants have the expectation that agents should prefer cooperation over individual actions, Study 2 should show different results.

Study 3 (*N* = 24) was carried out in order to test whether infants perform a similar utility calculus about instrumental action costs as in Study 1, even when their individual actions form part of a joint action performed by two cooperating individuals (see Figure 1E). This would allow us to answer the question whether infants’ expectation of a preference for cooperation demonstrated in Study 2 was grounded in computations of a utility calculus or not. As in Study 1, infants were presented with agents facing two routes that required them to move obstacles out of the way to reach their goal. Crucially however, each route was blocked by a single obstacle, thus requiring the agents to act jointly to move them away in both cases. The obstacles were formed either by a larger block that required each agent to perform three actions (Joint Costly condition) to push it away or by a smaller block, which required only two actions by each agent (Joint Easy condition). The smaller obstacle was identical to the combined volume of the two obstacles in the Individual condition of Study 2 and the Easy condition of Study 1. The alternative route involved a larger block that was identical in volume to that of the obstacle presented in the Joint condition of Study 2 and to the combined volume of the two obstacles used in the Costly condition of Study 1 (see Figure 1F). If infants perform an analysis of instrumental costs when the agents’ individual actions are part of the joint action performed by the two agents together, we expect to see the same pattern of looking times as in Study 1 with infants’ expectations being violated in the case when the agents choose the costlier path. If, however, infants do not show a differential expectation about which path is preferable, this would suggest that they have not computed the difference in instrumental costs when actions were performed as part of a joint action.

## RESULTS

### Study 1

**Figure 2.**
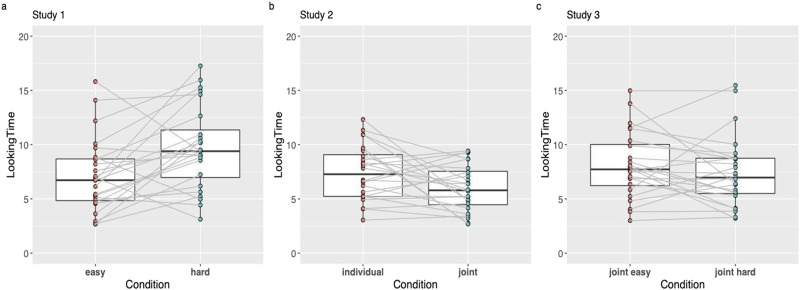
**Boxplots of within-subjects average looking time in Study 1, Study 2 and Study 3.** Horizontal lines in the boxplots indicate medians, and the bottom and top edges of the box indicate the 25th and 75th percentiles. Whiskers indicate the most extreme data points, not considered outliers (more than 2.5 standard deviations). Black points connected across boxes with light grey lines indicate mean looking times of individual participants in each condition (within-subjects). Statistical analyses are reported in the text.

Analysis of infants’ average looking times, calculated across the repetitions of videos within each condition, revealed that infants looked significantly longer in the Costly than the Easy condition (ANOVA with condition as within-subjects factor: *M*_costly_ = 10.329, CI_costly_ = 8.72–11.9; *M*_easy_ = 8.072, CI_easy_ = 6.46–9.67; *F*(1, 22) = 6.502, *p* = .018, *η*_p_^2^ = 0.228, see Figure 2A). These results indicate that infants expected the protagonist agent to choose the less costly action alternative of achieving the same goal. They also demonstrate that infants are sensitive to the agents’ individual instrumental utility and expect them to choose the action alternative that allows them to minimize the costs they have to incur to achieve their rewards, as predicted by the computations of instrumental utility calculus.

### Study 2

Analysis of infants’ average looking times, comparing Joint and Individual conditions, revealed that infants looked significantly longer in the Individual than in the Joint condition (ANOVA with condition as within-subjects factor: *M*_individual_ = 7.46; CI = 6.50–8.42; *M*_joint_ = 5.97, CI = 5.01–6.39; *F*(1, 23) = 5.8, *p* = .024, *η*_p_^2^ = 0.202, see Figure 2B). These results indicate that infants expected the protagonist agent to choose the Joint (despite the fact that it was instrumentally more costly) over the Individual (and instrumentally easier) course of action.

### Study 3

Analysis of infants’ average looking times, comparing Joint Costly and Joint Easy conditions, did not reveal a significant difference between the two conditions in infants looking times (ANOVA with condition as within-subjects factor: *M*_JoinEasyl_ = 8.08; *M*_JointCostly_ = 7.46; *F*(1, 23) = 1.06, *p* = 0.3, see Figure 2C). These results do not support the hypothesis that infants compute agents’ individual instrumental costs when agents are choosing between harder vs easier joint action routes to their goal (in contrast to Study 1).

## DISCUSSION

One of the defining features of human social life is the wide range of cooperative activities that we engage in. According to the principles of individual action planning, agents should aim to achieve any goal by incurring minimal possible costs. However, humans seem to often violate the principle of instrumental utility maximization when they choose to cooperate with conspecifics without direct apparent benefits. In fact, it has been shown that humans tend to prefer to act cooperatively even in situations in which they have to incur extra costs when compared to pursuing their goal alone (Curioni et al., 2022). Such decisions may reflect a special human motivation to cooperate with conspecifics. In line with this, evidence shows that even preschoolers seem to find a task more motivating and persist longer at it when it is framed in collaborative terms (Butler & Walton, 2013). Arguably, a preference for cooperation is adaptive as its early ontogenetic emergence might support the development of the extraordinary cooperative abilities that distinguish humans from other species (Melis & Semmann, 2010; Tomasello, 2009). With the current set of studies we demonstrate that infants at 14 months of age expect agents to choose to act together with a partner rather than achieving a goal individually, in a scenario where acting together is not necessary for achieving the goal (Study 2). Our results also demonstrate that even though infants are sensitive to the relative instrumental action costs when comparing two individual actions (Study 1), they do not seem to engage in the same utility analysis for actions embedded in a cooperative scenario (Study 3).

Specifically, Study 1 tested infants’ sensitivity to instrumental utility by comparing individual parallel actions differing in terms of the agents’ utility. The results demonstrated that infants could evaluate instrumental costs in the scenario we devised and expect agents to make instrumentally rational choices, in line with studies showing that infants expect agents to be utility maximisers (Liu et al., 2017; Liu & Spelke, 2017). Crucially, when a different group of infants, in Study 2, saw an identical scenario in terms of instrumental costs and rewards, but one of the alternative routes allowed the agents to remove the obstacle through cooperative action, infants expected the agents to choose to work together even though more effort was exerted by each agent to achieve the same reward. This result indicates that infants expect agents to have a preference to act cooperatively even in a scenario where cooperation is not necessary to achieve a certain goal (non-mutualistic). It also raised a new question of whether infants may ascribe an additional reward value (or reduced cost) to cooperative actions that goes beyond the observable instrumental costs and rewards of the actions agents perform. This could constitute the computational justification for the agents’ decision to preferentially engage in cooperation in this scenario. Alternatively, infants’ expectations may not be based on utility calculation of individual actions embedded in a joint action but may instead result from a different type of computation or heuristic, that is, one that dictates to engage in a cooperative activity whenever possible. To address these alternative explanations, Study 3 tested infants’ sensitivity to instrumental action costs in a joint action scenario. Here we did not find support for the hypothesis that infants apply the same utility calculations to joint cooperative actions. One possibility is that the utility calculations are suspended entirely in the joint context at this age; an alternative is that a different type of calculus is applied where instrumental costs and rewards are weighted and processed differently. Furthermore, we acknowledge that the two joint actions (easy vs. costly = 2 vs. 3 pushes) differed in the number of actions performed on the object compared to Study 1 (1 vs. 3 pushes). It is, therefore, possible that infants needed to see a larger difference in the number of actions between conditions to detect the difference in action costs.

Our findings support the hypothesis that infants expect agents to act together when presented with the choice between individual and joint actions. Such expectations may originate in a propensity to see cooperation as valuable, beyond its instrumental utility. Because in the present scenario the desired goal *could* be achieved by the agents individually, infants could not interpret the agent’s choice to cooperate by ascribing a preference for mutualistic collaboration—a type of cooperative activity that individuals engage in when they cannot achieve a goal on their own, and which numerous other species, including great apes, are capable of and willing to engage in (Tomasello et al., 2012). On the contrary, in our scenario, there is no observable necessity nor advantage to cooperating, therefore the agent’s decision to engage in cooperation is *not strictly utilitarian*. Arguably, such a scenario illustrates a paradigmatic case of preference for cooperation *per se*: agents engage in joint cooperative activity without any obvious prospect of immediate instrumental benefit from doing so.

Although here we did not find support for the hypothesis that infants apply the same utility calculations to individual and joint cooperative actions, this does not rule out the possibility that infants expect agents to apply utility calculus in joint contexts. It is plausible that such calculations do take place, but may be based on different parameters. For example, agents may assign additional value to performing actions together, which may contribute to, or override, the computation of instrumental costs. This interpretation is in line with findings from adult research. When comparing two joint action alternatives, adult individuals have been shown to compute the utility of joint actions and choose the most efficient alternative (Török et al., 2019). However, when choosing between individual and joint actions, adults prefer to perform joint over individual actions, despite the higher costs involved in joint actions (Curioni et al., 2022). Combined, these results from adult studies suggest that even when adult individuals are able to calculate the relevant instrumental costs of joint actions, their action choices cannot always be explained based on instrumental cost computation alone. Thus, the lack of difference in infants’ looking time in Experiment 3 leaves open the question of whether infants apply utility calculus to joint actions, what parameters are included in these computations, what parameters are included in these computations and whether, or how, these computations may change over development.

Importantly, engaging in cooperation constitutes a non-trivial challenge for the human cognitive system (Vesper et al., 2010). Successful cooperation through coordination would not be possible if humans did not master a wide range of dedicated cognitive skills, from mentalizing to representing conflicting perspectives (Moll et al., 2013), predicting and monitoring the behaviors of one’s social partners (Loehr et al., 2013), and representing the partner’s task and complementary actions for achieving shared goals (Newman-Norlund et al., 2007; Sebanz et al., 2003). In fact, when instrumental reward is kept constant, achieving a goal alone can be significantly less demanding than achieving it through coordination with a partner. However, as shown by Curioni et al. (2022), adults’ decision making in cooperative scenarios can’t be explained in terms of instrumental utility calculus alone. Our results indicate that infants’ expectations about agents’ behavior in the current study are coherent with adults’ preferences for cooperation. However, infants’ expectations seem to diverge from adults’ behavior in case of the choice between the less and more efficient joint action: while recent findings demonstrate that adults prefer more efficient joint means for goal achievement (Török et al., 2019, 2021), in our study we do not find a similar expectation in a comparable scenario. In fact, when directly testing whether infants evaluate the difference in instrumental costs of alternative joint actions (Study 3), we do not find evidence that they apply utility calculus to actions when they are part of a joint action. One possibility is that the ability to integrate and assess the instrumental costs in joint scenarios develops later in life. Alternatively, infants did not detect the difference in instrumental costs in this case in particular, as the difference was minimal. The processing of joint action may have required them to pay attention and integrate additional factors into their computations (e.g., the spatio-temporal cues of coordination) that are not present when observing individual actions alone, but which makes the minimal difference in instrumental costs that we presented to them in the joint action context insufficient to discriminate between the alternative routes. This, however, leaves an open question of why such minimal differences were sufficient for infants to discriminate between more and less costly individual actions in Experiment 1.

Current study has important limitations. In order to implement the differences in cost of actions presented to the infants, our stimuli contained necessary low-level visual differences that could influence infants’ perception and looking behaviour. In Experiment 1, agents in Costly and Easy conditions interacted with the obstacles a different number of times, and the objects differed in size. In Experiment 2, agents in Joint and Individual conditions interacted with different numbers and sizes of objects (1 big object vs 2 smaller objects), and objects in Individual condition were separated by a barrier, whereas no barrier was present in Joint condition. In Experiment 3, objects of the two joint conditions differed in size, and agents interacted with them different numbers of times. These low-level differences in visual complexity could arguably affect infants’ looking behaviour. However, to explain our results, different visual aspects of the scenes would have to drive the result. In particular, in Experiment 1, the number of actions would be a driving factor; however, in Experiment 2, the number of actions predicts the opposite result, and the driver in this case would have to be the number of objects. While we believe the more parsimonious explanation is that infants considered the two scenarios to differ based on the jointness vs. individual nature of the actions they observed (explaining both sets results), we acknowledge that these perceptual differences represent a potential confound.

The current studies present the initial steps in the investigation of infants’ utility computation of cooperative goal achievement, which is still largely uncharted territory for both infant and adult cognition research. Further research is needed to explore and shed light on the computational mechanisms involved in evaluating the expected rewards and costs assigned to cooperative actions.

We propose that a preference for cooperation may have several implications for human adults’ and children’s fitness. We speculate that such a motivational mechanism could serve the function of prompting spontaneous engagement in cooperative activities in a broad variety of contexts and from an early age. By reducing the primacy of instrumental utility and cost aversion in the human cognitive system (Kool et al., 2010; Kurzban et al., 2013), it may increase the likelihood of individuals’ engagement in cooperative activities that would not necessarily be selected on the basis of instrumental benefit only. In turn, such readiness to engage in cooperative activities even without observable additional benefits may lead to several long-term benefits. Firstly, frequent engagement in cooperation from early on in ontogeny can provide unique learning opportunities, where individuals can acquire skills and abilities that are crucial to develop proficiency in cooperation. This would ensure that individuals would be able to harness the large benefits that cooperative ventures yield. Secondly, individuals engaging in cooperative activities with several different partners would gain opportunities to learn about partners’ cooperative skills and relevant dispositional properties (such as trustworthiness and commitment), which could inform future partner choice. Lastly, this could serve the signaling function of an agents’ availability for and willingness to engage in cooperative interactions.

One likely distal cause of human preference for cooperation is the potential (and future) social benefit of cooperative engagement. In line with this, evidence from a study by Tan and Hare (2013) shows that bonobos are willing to share their food resources with strangers, as they anticipate the future benefits of establishing new friendships and expanding their social network. However, even assuming that long term (non-observable) benefits may distally cause human’s preference for cooperative behavior, we argue that infants’ expectations about agents’ choice to act together are likely rooted also in the analysis of the proximal causes of agents’ observed decisions in the particular situation.

Finally, the present results pose fundamental questions for future research.

The underlying cognitive processes that support infants’ expectations about cooperative preferences remain to be explored. Our studies indicate that, when presented with evidence of instrumental actions’ costs in a non-social scenario, infants can identify the action alternative that maximizes individual utility and expect other agents to choose this alternative. When comparing joint versus individual actions, infants expect agents to prefer the cooperative action alternative even though it requires agents to exert more effort. However, when presented with differential instrumental costs in two separate joint action scenarios, infants do not show an expectation of cost reduction similar to an individual action scenario. We do not know if such a disparity is due to infants’ inability to detect the differences in instrumental costs in the particular cases of joint action scenarios presented in our studies or whether infants compute instrumental costs of joint actions differently.

Relatedly, it is an open question whether infants represent and integrate the instrumental and social utility of actions. On the one hand, instrumental costs and benefits of actions may seem more directly comparable, as they can be interpreted by infants in terms of physical principles (Téglás et al., 2011) and can be mapped onto action experience (although there are no clear-cut answers as to the contribution of experience in action understanding, see Ullman et al., 2017). On the other hand, our results do not provide evidence that the process of quantification and comparison of rewards and costs of individual actions when embedded in cooperative actions is similar to the utility analysis applied to individual action scenarios. This poses the further challenge to identify whether there is a common currency used to compute the utility of instrumental actions in social contexts. One possibility is that social costs and benefits of actions are represented in a format that allows for direct integration into the utility calculation of cooperation. This would imply that infants can compute and compare different kinds of utilities associated with a given action on a common scale. Alternatively, the action context may determine the rules governing utility calculations. Once a particular type of action context is identified (e.g., “cooperation”), a dedicated utility scale would apply. By testing what instrumental costs infants would allow for a cooperative action to be preferable over individual goal achievement, it would be possible to address fundamental questions about the nature of infants’ representation of cooperative actions and their utility.

## METHODS

### Procedure and Apparatus

Infants were tested in a quiet, dimly lit room, approximately 65 cm away from the computer monitor. They sat on their caregiver’s lap. The caregiver wore darkened glasses, so that they could not see the stimuli. They were instructed not to interact with the child in any verbal or non-verbal manner, unless the infant turned completely away from the stimulus for more than 5 s, in which case the caregiver was instructed to turn them back in the initial position facing the screen. This procedure ensured that infants could freely turn away when they were not interested in the display but could be reoriented for the next trial without interfering with the triggering of a timeout. Infants saw 8 test trial movies, 4 of Joint Action condition and 4 of Individual Parallel condition. The last frame of each test movie remained still on the screen until the infant looked away for more than 2 consecutive seconds, or else looked at the screen for more than 30 cumulative seconds. The experimenter monitored infants via the camera placed above the screen and controlled the advancement of trials from a separate room. The recordings were further analysed offline.

The movies were designed as 3D animations in Autodesk Maya 2018. They were then exported as movies at 25 fps and further edited with QuickTime Player 7 software. The experiment was run on an Apple Mac Pro Quad Core 2.8 computer, controlled by PsyScope X. The stimuli appeared on a 24-inch TFT screen.

To achieve sufficient power for effect sizes of 0.6 and higher with the looking time measure, samples of 20 or 24 infants suffice (Oakes, 2017). We chose the sample of 24 participants per study due to our counterbalancing scheme.

The studies employed the violation of expectation looking time paradigm according to which infants tend to look longer at unexpected events (for a review see Aslin, 2007). The studies employed the violation of expectation looking time paradigm, according to which infants tend to look longer at unexpected events (for a review, see Aslin, 2007). However, we took an unconventional approach and presented infants with test trials that were not preceded by familiarization. Our rationale was to avoid priming infants with different degrees of action efficiency and, instead, to register their spontaneous expectations in the experimental scenario.

It is important to point out that, unlike the habituation paradigm, the Violation of Expectation (VoE) does not rely on familiar vs. novel stimuli but on unexpected events—those that infants do not predict. At 15 months, infants possess a rich set of expectations about both the physical and social aspects of the world, including expectations about how agents are likely to act. With the current design, our goal was to tap into infants’ spontaneous expectations about the world, agents, and events within it.

By eliminating familiarization we aimed to avoid biasing infants toward a) either Joint or Individual action paths and b) individual agents’ preferences for either. Additionally, we aimed to refrain from demonstrating actions that did not follow the rationality principle (e.g., if during familiarization agents performed the same actions but without barriers present—in this case, the rational action would be taking the shortest path available and going straight to the apples without moving the obstacles).

The order of the conditions counterbalancing was ABABABAB; which condition was displayed first was counterbalanced across participants.

### Stimuli

#### Study 1.

The scene depicted an assembly of horizontally positioned blocks and barriers in the middle of the scene, with two agents (blue cylinder and yellow cone) in front of the barriers, and two red spherical objects—“apples”—behind them (see Figure 1A and B). Two groups of blocks on each side of the screen (left and right) constituted two alternative paths for the agents to reach the goal objects on the other side of the barriers. In Study 1 the action paths were Individual actions that differed in instrumental utility (Easy and Costly condition). Infants saw 8 movies, 4 Individual Easy and 4 Individual Costly condition trials.

On either right or left (counterbalanced) of the midline of the scene, there were two big blocks (Costly) and on the opposite side two smaller blocks divided by a barrier (Easy). Crucially, the two blocks in the Costly condition were 30% larger in volume than the sum of the two small blocks in the Easy condition. The videos depict the agents pushing either two smaller blocks or two big blocks individually. Once the obstacles have been cleared, they proceed towards the red spherical objects and ‘eat’ them.

Each trial started with the protagonist agent moving first, turning left and right, as if inspecting the options. After that, the protagonist started to move towards one of the two sides of the scene (choosing the means to achieve the goal, either Easy or Costly Action), while the second agent followed.

In Easy trials each agent arrived in front of one of the small blocks and moved it away with one long push. Once the blocks were pushed away, the two agents moved around them, reached their goal object (apple), and ate it. Onset and offset of the two agents’ actions (pushing the block, and the approach of the goal object) were desynchronized by 1 second, so as to stress the independence of the two actions and to prevent that they would reach their goal object at the same time.

In Costly trials, both agents arrived in front of one of the big blocks and moved it away with 3 pushes. Once the block was pushed away, the two agents moved around it, reached their goal object (apple) and ate it. Crucially, in this condition both agents had to perform more actions to move the block away (which is understood by infants as a measure of effort; Scott & Baillargeon, 2013), therefore making this condition instrumentally more costly.

In both conditions, the time to push the obstacles and to achieve the goal was the same. Moreover, in both conditions the agents reached their goal object at different times. This was made to ensure that infants could not attribute to the agent the goal of reaching the apples and eating them together. Side of Easy and Costly route options, and side of the protagonist relative to the follower (left/right) during the pushing actions, was counterbalanced across trials. This ensured that the order of agents’ arrival at the goal object was also counterbalanced.

#### Study 2.

Infants saw 8 test trials, half of them depicting a Joint Action, and half an Individual Parallel action. The Individual Parallel condition is identical to the Easy condition in Study 1 (one long push required to move the blocks). The Joint Action condition is equivalent in terms of the protagonist’s relative utility to the Costly condition in Study 1 (see Figure 1C and D).

On either right or left (counterbalanced) of the midline of the scene, there was one big block (Joint Action) and on the opposite side two smaller blocks divided by a barrier (Individual Parallel). Crucially, the single big block in the Joint Action condition was 30% larger in volume than the sum of the two small blocks in the Individual Parallel condition. The videos depict the agents pushing either two smaller blocks individually or one bigger block in coordination with their partner and, once the obstacles have been cleared, they proceed towards the red spherical objects and ‘eat’ them.

#### Individual Parallel Trials Were Identical to Easy Trials in Study 1.

In Joint trials, both agents arrived in front of the same big block: initially, each agent made a short push from the sides, after which they twice pushed the block simultaneously. Once the block was pushed away, the two agents moved around it, reached their goal object (apple), and ate it.

This condition is equivalent in terms of the protagonist’s relative utility to the Costly condition in Study 1. All the movement properties of the agents in both conditions were identical to the two conditions of Study 1. The only crucial difference between the Costly condition and the Joint Action condition in Study 2 is that agents in the Joint Action condition acted on a single block together, therefore performed one individual and two coordinated actions.

#### Study 3.

Infants saw eight trials, half of them belonging to the Costly Joint condition, and half to the Easy Joint condition. The setup was the same as in the previous experiments, with the exception that the obstacles on both sides consisted of a single block (see Figure 1E and F). Similar to the stimuli used in the preceding studies, one block was about 30% bigger than another. The Costly Joint condition was identical to the Joint Action condition of Study 2. In the Easy Joint condition agents removed the smaller block pushing it once acting simultaneously together following an attempt by each of them to remove it alone. Thus, the crucial difference between the conditions was the instrumental cost of moving the blocks (smaller size vs bigger size; two pushes for each agent vs three pushes for each agent), but in both conditions agents had to act together.

### Participants

Participants were recruited from the larger Budapest area in Hungary. Parents signed a written informed consent before taking part in the study, and all participants received a small toy as a gift for their participation. This research complied with relevant ethical regulations and was approved by the Hungarian Ethical Review Committee for Research in Psychology (EPKEB).

#### Study 1.

Final sample included twenty-four full-term 14-month-old infants (10 females, mean age = 14 months 11 days, range 14 m 9 d to 15 m 25 d) from Hungarian-speaking families, with no visual or hearing problems. 3 additional infants were tested but excluded from the final sample: 2 due to fussiness and 1 for parental intervention.

#### Study 2.

Final sample included twenty-four full-term 14-month-old infants (11 females, mean age = 14 months 21 days, range 14 m 0 d to 15 m 10 d) from Hungarian-speaking families, with no visual or hearing problems. Six infants were tested but excluded from the final sample: 4 due to fussiness and 2 due to equipment failure.

#### Study 3.

Final sample included twenty-four full-term 14-month-old infants (14 females, mean age = 14 months 26 days, range 13 m 29 d to 15 m 15 d) from Hungarian-speaking families, with no visual or hearing problems. Seven additional infants were tested but excluded from the final sample: 4 due to fussiness and 3 for not contributing enough data.

### Coding

Looking time was calculated based on off-line coding. Measuring looking time started after the agents stopped moving, at the end of the video. Half of the sample (24 infants) was randomly selected and coded by the second coder. The inter-coder agreement was high, with correlational coefficient of 0.96 in Study 1, 0.95 in Study 2 and 0.91 in Study 3.

### Data Treatment

The data was tested for normality (after eliminating outliers, performed on mean values per condition per participant) using the Shapiro-Wilks test and did not show any significant deviation from normality in all groups. Data from an infant was included in the analysis if at least 2 trials of each condition have been contributed. A trial was rejected if infants looked for less than 2 or more than 30 cumulative seconds, or was outside of 2.5 standard deviations.

## ACKNOWLEDGMENTS

This research was supported by the European Research Council (ERC) under the European Union’s Seventh Framework Programme [FP7/2007-2013/ERC Grant 609819], project SOMICS.

## AUTHOR CONTRIBUTIONS

L.V., K.B., G.G., G.K., A.C.: Conceptualization; L.V., A.C.: Formal Analysis; G.G., G.K.: Funding Acquisition; L.V.: Investigation; L.V., A.C.: Writing – Original Draft; K.B., G.G., G.K.: Writing – Review & Editing.

## FUNDING INFORMATION

This research was supported by the European Research Council (ERC) under the European Union’s Seventh Framework Programme [FP7/2007-2013/ERC Grant 609819], project SOMICS. The funders had no role in study design, data collection and analysis, decision to publish, or preparation of the manuscript.

## DATA AVAILABILITY STATEMENT

The data that support the findings of this study (looking time) are available from the corresponding author upon request. The raw data (video recordings) are not available due to the sensitive nature and them containing information that compromises research participant privacy/consent.
